# Sec12 Binds to Sec16 at Transitional ER Sites

**DOI:** 10.1371/journal.pone.0031156

**Published:** 2012-02-08

**Authors:** Elisabeth A. Montegna, Madhura Bhave, Yang Liu, Dibyendu Bhattacharyya, Benjamin S. Glick

**Affiliations:** 1 Department of Molecular Genetics and Cell Biology, The University of Chicago, Chicago, Illinois, United States of America; 2 Advanced Centre for Treatment Research and Education in Cancer (ACTREC), Tata Memorial Centre, Kharghar, Navi Mumbai, India; Institute of Molecular and Cell Biology, Singapore

## Abstract

COPII vesicles bud from an ER domain known as the transitional ER (tER). Assembly of the COPII coat is initiated by the transmembrane guanine nucleotide exchange factor Sec12. In the budding yeast *Pichia pastoris*, Sec12 is concentrated at tER sites. Previously, we found that the tER localization of *P. pastoris* Sec12 requires a saturable binding partner. We now show that this binding partner is Sec16, a peripheral membrane protein that functions in ER export and tER organization. One line of evidence is that overexpression of Sec12 delocalizes Sec12 to the general ER, but simultaneous overexpression of Sec16 retains overexpressed Sec12 at tER sites. Additionally, when *P. pastoris* Sec12 is expressed in *S. cerevisiae*, the exogenous Sec12 localizes to the general ER, but when *P. pastoris* Sec16 is expressed in the same cells, the exogenous Sec12 is recruited to tER sites. In both of these experimental systems, the ability of Sec16 to recruit Sec12 to tER sites is abolished by deleting a C-terminal fragment of Sec16. Biochemical experiments confirm that this C-terminal fragment of Sec16 binds to the cytosolic domain of Sec12. Similarly, we demonstrate that human Sec12 is concentrated at tER sites, likely due to association with a C-terminal fragment of Sec16A. These results suggest that a Sec12–Sec16 interaction has a conserved role in ER export.

## Introduction

In the secretory pathway, newly synthesized proteins are exported from the ER in COPII coated transport vesicles [1], [2], [3]. COPII vesicles bud from ribosome-free ER domains known as transitional ER (tER) sites or ER exit sites [4], [5], [6]. The mechanism that generates tER sites is unknown, but our working model is that tER sites form by a self-organization process that depends on the specific properties of components involved in COPII assembly [7], [8]. Therefore, an analysis of tER site formation must build on knowledge of how COPII coat proteins interact with each other and with partner proteins.

The first step in COPII coat assembly is the exchange of GDP for GTP on the small GTPase Sar1. This reaction is catalyzed by the guanine nucleotide exchange factor Sec12, which spans the ER membrane with its catalytic domain facing the cytosol [9]. Sar1-GTP associates with the ER membrane and recruits the Sec23/24 heterodimer. Sec23 acts as a GTPase activating protein for Sar1, while Sec24 functions to capture cargo into the nascent vesicle [2]. Sec23/24 binds the Sec13/31 heterodimer, which polymerizes to form the outer shell of the coat [10]. In all eukaryotes studied to date, Sec23/24 and Sec13/31 localize to punctate ER regions that operationally define tER sites [5].

By contrast, the localization of Sec12 is variable. In the budding yeast *Pichia pastoris*, Sec12 is concentrated at tER sites [11], [12], but in *Saccharomyces cerevisiae*, Sec12 is found throughout the ER [11], [13]. This variability suggests that Sec12 localization does not establish tER sites, and indeed, mutations that delocalize *P. pastoris* Sec12 (PpSec12) to the general ER do not prevent tER site formation [12]. Instead, PpSec12 is recruited to tER sites by a partner protein that interacts with the PpSec12 cytosolic domain [12]. Overexpression of PpSec12 results in localization to the general ER, indicating that binding by the partner protein is saturable [12]. Identification of the partner protein should help to clarify the functional significance of this novel Sec12 interaction.

A candidate for the PpSec12 partner protein is Sec16, a large peripheral ER membrane protein that functions in ER export and interacts with multiple COPII pathway components [14], [15], [16]. In previous work, we isolated a thermosensitive *P. pastoris* mutant with fragmented tER sites, and identified the cause of the defect as a missense mutation in *SEC16* [8]. Sec16 colocalizes with COPII coat proteins at tER sites in both *P. pastoris* and *S. cerevisiae* [8]. Mammalian and *Drosophila* cells also contain Sec16 homologs that localize to tER sites and play a key role in tER organization [17], [18], [19], [20], [21]. In the *P. pastoris sec16* strain at the nonpermissive temperature, most of the mutant Sec16 protein is displaced from tER sites, and most of the PpSec12 is delocalized to the general ER [8], consistent with the idea that *P. pastoris* Sec16 (PpSec16) recruits PpSec12 to tER sites.

Here, we demonstrate that a C-terminal fragment of PpSec16 binds to PpSec12, and that this interaction recruits PpSec12 to tER sites. Moreover, mammalian Sec12 is concentrated at tER sites, and the cytosolic domain of mammalian Sec12 can bind to a C-terminal fragment of Sec16A, which is the mammalian ortholog of yeast Sec16. Thus, the Sec12–Sec16 interaction may have a conserved role in generating COPII vesicles and tER sites.

## Results

### Overexpression of PpSec16 in *P. pastoris* restores tER localization to overexpressed PpSec12

**Figure 1:**
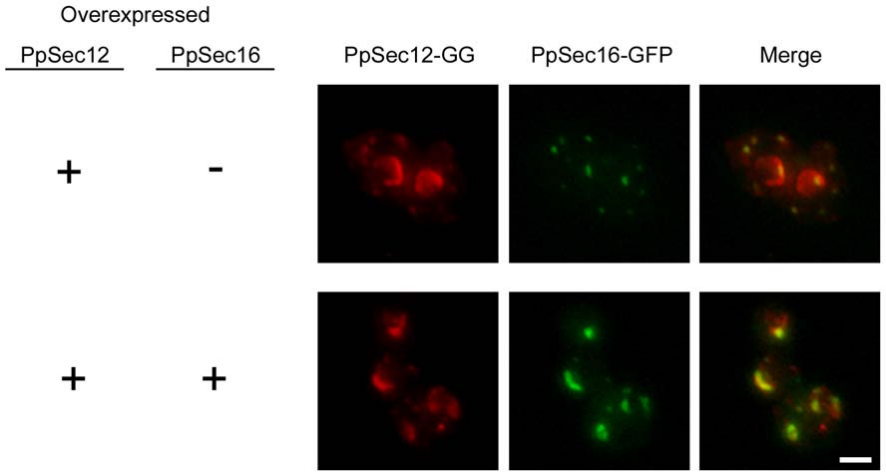
Recruitment of overexpressed PpSec12 to tER sites in *P. pastoris* by simultaneous overexpression of PpSec16. PpSec12 was tagged with the Glu-Glu epitope (PpSec12-GG) by gene replacement, and a second untagged copy of PpSec12 was expressed in the same cells using the methanol-inducible *AOX1* promoter, resulting in a high total level of PpSec12 expression. Top row: in a strain overexpressing PpSec12, PpSec16 was expressed at normal levels after being tagged by gene replacement with GFP. A small fraction of the PpSec12-GG colocalized with PpSec16-GFP, but most of the PpSec12-GG was in the general ER as indicated by the prominent nuclear envelope signal. Bottom row: in a strain overexpressing PpSec12, PpSec16-GFP was overexpressed as a second copy using the *AOX1* promoter. Most of the PpSec12-GG colocalized with PpSec16-GFP in exaggerated tER sites. Scale bar, 2 µm.

When Glu-Glu epitope-tagged PpSec12 (PpSec12-GG) was expressed at normal levels in *P. pastoris*, this protein localized to tER sites, but when untagged PpSec12 was overexpressed in the same cells, most of the PpSec12-GG molecules were found in the general ER [12]. This general ER pattern included partial or complete fluorescent rings representing the nuclear envelope (Fig. 1, top row). We infer that the PpSec12-GG molecules marked the distribution of the combined pool of tagged and untagged PpSec12 molecules, and that this combined pool had saturated the tER-localized partner protein.

Cells expressing PpSec12-GG and overexpressing PpSec12 were engineered to express GFP-tagged PpSec16 (PpSec16-GFP) at either normal or elevated levels. When PpSec16-GFP was expressed at normal levels, it localized to punctate tER sites that contained only a small fraction of the PpSec12-GG (Fig. 1, top row). However, when PpSec16-GFP was overexpressed, it was found in abnormally large punctate structures that also contained most of the PpSec12-GG (Fig. 1, bottom row). The large punctate structures generated by simultaneous overexpression of PpSec12 and PpSec16 were exaggerated tER sites because they also contained the COPII coat protein Sec13 (Fig. S1). These results are consistent with the idea that PpSec12 binds PpSec16, and that overexpressed PpSec16 recruits overexpressed PpSec12 to tER sites.

### PpSec16 can recruit PpSec12 to tER sites in *S. cerevisiae*

**Figure 2:**
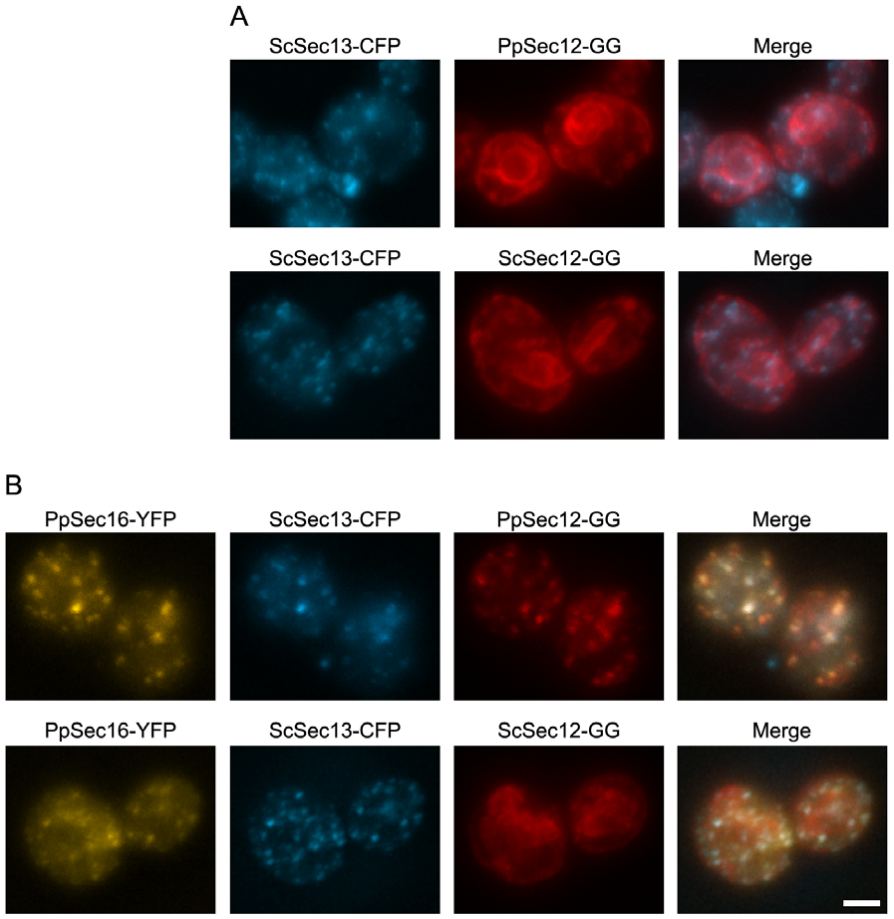
Recruitment of PpSec12 to tER sites in *S. cerevisiae* by simultaneous expression of PpSec16. (A) *S. cerevisiae* cells expressed *S. cerevisiae* Sec13-CFP (ScSec13-CFP) plus either PpSec12-GG (top row) or ScSec12-GG (bottom row). Both versions of Sec12 localized to the general ER. (B) Same as (A), except that the *S. cerevisiae* cells also expressed PpSec16-YFP under control of the inducible *GAL10* promoter. PpSec16-YFP colocalized with ScSec13-CFP at tER sites, and now tER localization was observed for PpSec12-GG but not for ScSec12-GG. Scale bar, 2 µm.

As a further test of whether PpSec16 can recruit PpSec12 to tER sites, we expressed both proteins in *S. cerevisiae*, which has numerous small tER sites [11], [22], [23]. When either PpSec12-GG or Glu-Glu epitope-tagged *S. cerevisiae* Sec12 (ScSec12-GG) was expressed alone in *S. cerevisiae*, the tagged Sec12 was found in the general ER (Fig. 2A) as previously observed [12]. When PpSec16-YFP was simultaneously expressed in *S. cerevisiae*, this protein colocalized with CFP-tagged *S. cerevisiae* Sec13 (ScSec13-CFP) at tER sites, and now PpSec12-GG was also concentrated at tER sites (Fig. 2B). By contrast, expression of PpSec16-YFP in *S. cerevisiae* did not change the localization of ScSec12-GG (Fig. 2B), indicating that the recruitment of PpSec12-GG to tER sites was due to a specific interaction with PpSec16-YFP.

### A C-terminal region of PpSec16 is required for PpSec12 localization

**Figure 3:**
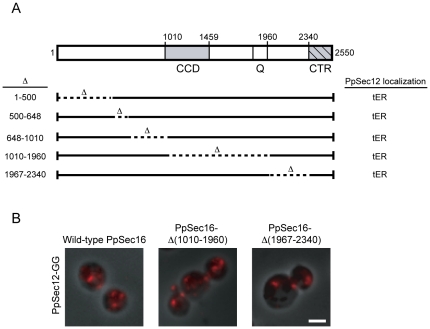
Effect of deleting nonessential PpSec16 regions on PpSec12 localization in *P. pastoris*. (A) Diagram of the domain organization of PpSec16. Shading indicates conserved regions while hatch marks indicate an essential region. CCD, central conserved domain; Q, glutamine-rich region; CTR, C-terminal conserved region. Deletions introduced by gene replacement are indicated. None of these deletions affected PpSec12 localization. (B) Representative images of PpSec12-GG localization in *P. pastoris* cells carrying the indicated deletions in PpSec16. Scale bar, 2 µm.

As shown in Fig. 3A, PpSec16 contains a central conserved domain (CCD) as well as a conserved C-terminal region (CTR) that is essential for life [8], [14]. To determine which parts of PpSec16 affect PpSec12 localization, we systematically deleted nonessential regions of PpSec16 by chromosomal gene replacement, and then examined PpSec12 localization in these cells. All of these strains showed normal tER localization of PpSec12-GG (Fig. 3A,B).

**Figure 4:**
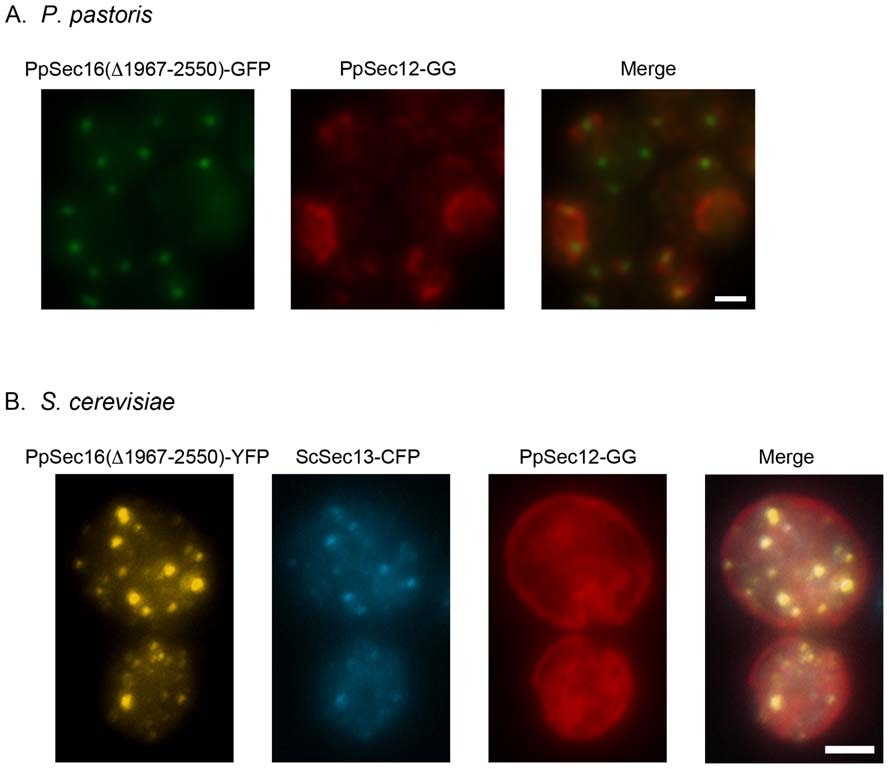
Requirement of the C-terminal portion of PpSec16 for tER localization of PpSec12 in both *P. pastoris* and *S. cerevisiae*. (A) As in Fig. 1, *P. pastoris* cells expressed PpSec12-GG from the endogenous promoter plus untagged Sec12 from the *AOX1* promoter, resulting in a high total level of PpSec12 expression. In the same cells, a truncated version of PpSec16 lacking residues 1967–2550 was tagged with GFP and overexpressed as a second copy using the *AOX1* promoter. PpSec16(Δ1967–2550)-GFP was found in punctate tER sites. By contrast to the result obtained when full-length PpSec16 was expressed (Fig. 1), PpSec12-GG was found in the general ER. (B) *S. cerevisiae* cells expressed the same truncated version of PpSec16 as in (A), except that the protein was tagged with YFP and was expressed under control of the *GAL10* promoter. As in Fig. 2B, ScSec13-CFP and PpSec12-GG were also expressed in these cells. By contrast to the result obtained when full-length PpSec16 was expressed (Fig. 2B), PpSec12-GG was found in the general ER. Scale bars, 2 µm.

*S. cerevisiae* and its close relatives contain not only Sec12, but also the Sec12-like protein Sed4, which interacts with a C-terminal fragment of *S. cerevisiae* Sec16 (ScSec16) [24], [25], [26]. Therefore, we suspected that PpSec12 might interact with a C-terminal fragment of PpSec16. To test this hypothesis, we repeated the experiment of simultaneously overexpressing PpSec12 and PpSec16 in *P. pastoris*, except that the overexpressed PpSec16 was truncated. This truncation removed residues 1967–2550, which encompassed the CTR plus a nonconserved stretch between the glutamine-rich region and the CTR. The truncated PpSec16 was found at tER sites, but unlike intact PpSec16 (see Fig. 1), the truncated PpSec16 was unable to restore tER localization to overexpressed PpSec12 (Fig. 4A). We also expressed the truncated version of PpSec16 in *S. cerevisiae* cells that simultaneously expressed PpSec12. Unlike intact PpSec16 (see Fig. 2B), the truncated PpSec16 was unable to recruit PpSec12 to tER sites (Fig. 4B). These results indicate that a C-terminal fragment of PpSec16 is needed for the tER localization of PpSec12.

**Figure 5:**
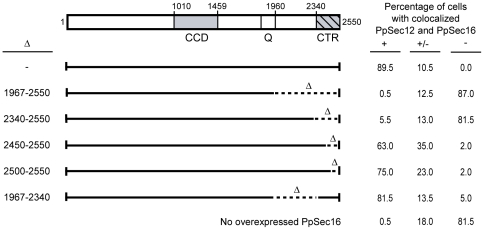
Requirement of the C-terminal portion of PpSec16 for recruiting overexpressed PpSec12 to tER sites. As in Fig. 1, PpSec16-GFP was overexpressed in *P. pastoris* cells overexpressing PpSec12, except that deletions were introduced as indicated near the C-terminus of PpSec16. Two hundred randomly chosen cells from each of the indicated *P. pastoris* strains were examined by immunofluorescence and scored for colocalization of PpSec12-GG with PpSec16-GFP. Cells in which nearly all of the PpSec12-GG overlapped with PpSec16-GFP were scored as having strong colocalization (+). Cells in which PpSec12-GG showed clear concentration in the PpSec16-GFP puncta but also showed prominent staining outside of these puncta were scored as having partial colocalization (+/−). Cells showing no visible concentration of PpSec12-GG in the PpSec16-GFP puncta were scored as having no colocalization (−). Colocalization was virtually abolished by deleting the entire C-terminal portion of PpSec16, and was strongly reduced by deleting only the C-terminal conserved region (CTR).

To determine which parts of the C-terminal fragment of PpSec16 interact with PpSec12, we made deletions within the C-terminal fragment, and then simultaneously overexpressed PpSec12 and a mutant PpSec16 in *P. pastoris*. Two hundred cells from each strain were scored to determine if PpSec12 was strongly colocalized with PpSec16 at tER sites, or partially colocalized, or not colocalized (Fig. 5). In the control strain overexpressing full-length PpSec16, ∼90% of the cells showed strong colocalization of PpSec12 with PpSec16. Deletion of the entire C-terminal fragment of PpSec16 resulted in <1% of the cells showing strong colocalization. Deletion of the nonconserved stretch (residues 1967–2340) had a mild effect, with ∼80% of the cells showing strong colocalization, indicating that the nonconserved stretch within the C-terminal fragment plays some role in recruiting PpSec12 to tER sites. Deletion of the CTR (residues 2340–2550) had a dramatic effect, with only ∼5% of the cells showing strong colocalization, indicating that the CTR plays a major role in recruiting PpSec12 to tER sites. Smaller deletions that truncated the CTR had intermediate effects. These results indicate that recruitment of PpSec12 to tER sites requires a C-terminal fragment of PpSec16, and that the bulk of this interaction is mediated by the CTR.

### A C-terminal fragment of PpSec16 binds the cytosolic domain of PpSec12 *in vitro*

**Figure 6:**
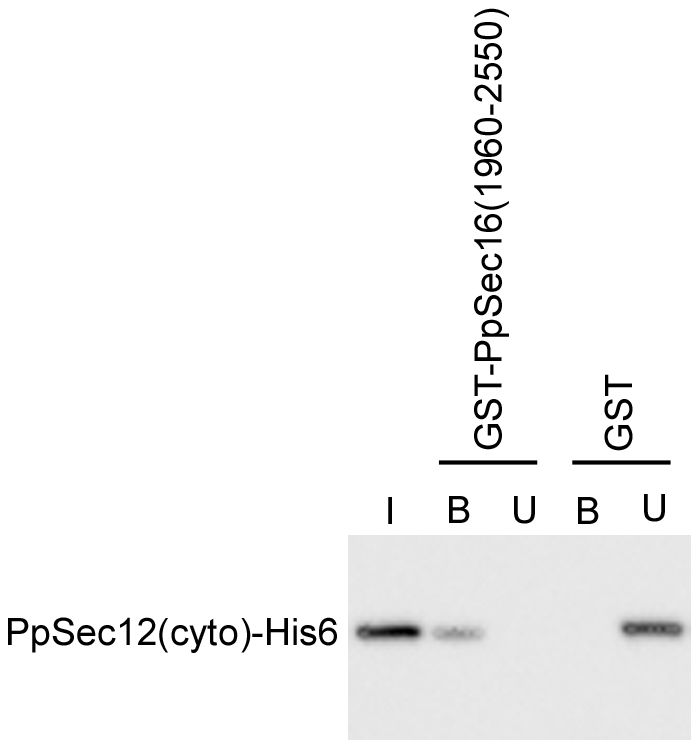
Biochemical interaction of the C-terminal portion of PpSec16 with the cytosolic domain of PpSec12. Glutathione-agarose beads were incubated with a bacterial lysate from cells expressing either GST alone, or GST fused to the C-terminal residues 1960–2550 of PpSec16. Sufficient lysate was used to saturate the binding sites on the glutathione-agarose. A second incubation was then performed with sub-saturating amounts of a bacterial lysate from cells expressing a hexahistidine-tagged version of the cytosolic domain of PpSec12 (PpSec12(cyto)-His6). The beads were centrifuged, and the unbound material in the supernatant was collected. Bound protein was eluted from the beads with 100 mM glutathione. I, input (100% relative to other lanes); U, unbound; B, bound. PpSec12(cyto)-His6 bound to the beads carrying GST-PpSec16(1960–2550) but not to the beads carrying GST alone.

To determine if a C-terminal fragment of PpSec16 interacts directly with the cytosolic domain of PpSec12, we used bacterial expression to produce glutathione S-transferase (GST) fused to residues 1960–2550 of PpSec16. In parallel, we used bacterial expression to produce the cytosolic domain of PpSec12 with a C-terminal hexahistidine tag. Glutathione-agarose beads were incubated with a lysate from cells expressing either GST alone or GST-Sec16(1960–2550), and were subsequently incubated with a lysate from cells expressing PpSec12(cyto)-His6 (Fig. 6, “I”). The unbound material was collected (Fig. 6, “U”), and the bound protein (Fig. 6, “B”) was eluted from the beads with glutathione. With GST alone, all of the PpSec12(cyto)-His6 was in the unbound fraction. With GST-PpSec16(1960–2550), none of the PpSec12(cyto)-His6 was in the unbound fraction and a significant amount could be eluted from the beads with glutathione. The remaining PpSec12(cyto)-His6 that had bound to GST-PpSec16(1960–2550) was apparently lost during the wash steps (data not shown), suggesting that this binding is readily reversible. These data provide evidence for a direct interaction between a C-terminal fragment of PpSec16 and the cytosolic domain of PpSec12.

### Viability of *S. cerevisiae* does not require strong interaction of a Sec12 family member with Sec16

PpSec12 binds to the CTR of PpSec16, and Sed4 binds to the CTR of ScSec16, and the CTR is essential for life, so we wondered whether the interaction of a Sec12 family member with Sec16 is essential. To answer this question, we took advantage of the finding that PpSec12 can replace ScSec12 in *S. cerevisiae* [12] even though PpSec12 binds to ScSec16 weakly or not at all (see above). The earlier Sec12 replacement was performed with a *SED4* strain of *S. cerevisiae*, but if PpSec12 replaced ScSec12 in a *sed4*Δ strain, there would be no strong interaction of a Sec12 family member with ScSec16. Therefore, the crucial question is whether PpSec12 can still replace ScSec12 in a *sed4*Δ strain of *S. cerevisiae*.

**Figure 7:**
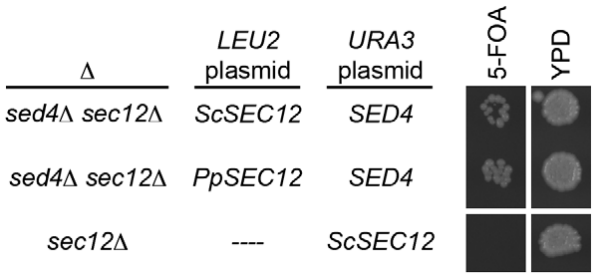
Viability of *S. cerevisiae* cells carrying *PpSEC12* as the only gene from the *SEC12* family. A plasmid shuffle was performed in *sed4*Δ *sec12*Δ cells, with *SED4* in a *URA3* plasmid plus either *ScSEC12* (top row) or *PpSEC12* (middle row) in a *LEU2* plasmid. Both strains grew on rich media (YPD) and also on media containing 5-FOA, indicating that *PpSEC12* could replace *ScSEC12* even in the absence of *SED4*. As a control, *sec12*Δ cells carrying *ScSEC12* on a *URA3* plasmid were plated on the same media, and no growth was seen in the presence of 5-FOA.

This experiment was performed using a plasmid shuffle approach. The chromosomal *SED4* and *SEC12* genes were deleted, and were replaced using two plasmids: a *URA3* plasmid carrying *SED4*, and a *LEU2* plasmid carrying either *ScSEC12* or *PpSEC12*. 5-fluoroorotic acid (5-FOA) was then used to select for cells that had lost the *URA3 SED4* plasmid. The cells that had lost the *URA3 SED4* plasmid were viable regardless of whether the *LEU2* plasmid carried *ScSEC12* or *PpSEC12* (Fig. 7). Thus, PpSec12 can replace ScSec12 even in a *sed4*Δ strain of *S. cerevisiae*, indicating that a strong interaction of a Sec12 family member with Sec16 is not essential for life in this yeast.

### Human Sec12 localizes to tER sites and binds a C-terminal fragment of Sec16A

**Figure 8:**
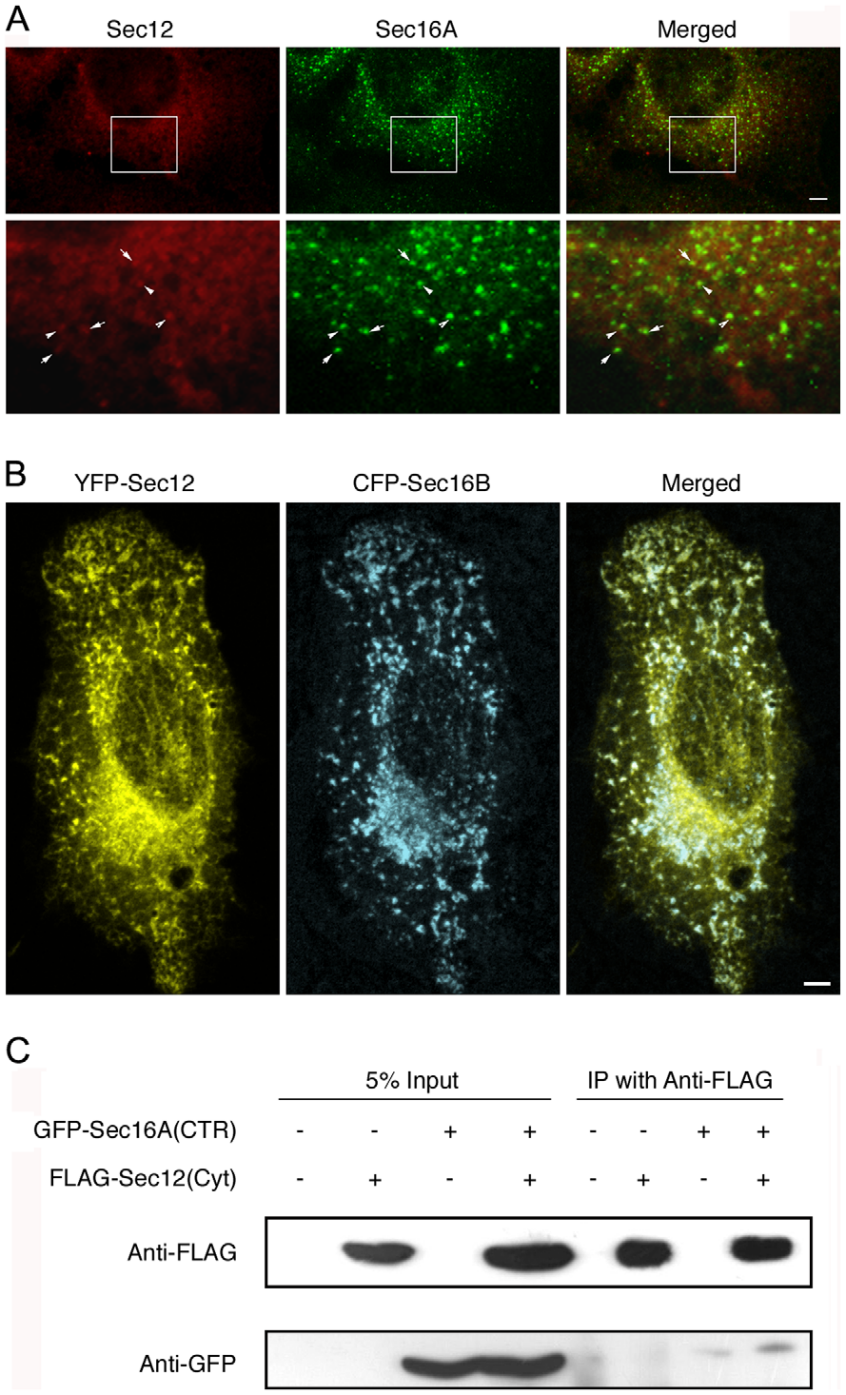
Colocalization of mammalian Sec12 with Sec16A at tER sites. (A) U2OS human osteosarcoma cells were subjected to immunofluorescence as described [28] using commercial antibodies against human Sec12 and Sec16A. Scale bar, 2 µm. (B) Plasmids encoding YFP-tagged full-length human Sec12 and CFP-tagged full-length Sec16B were co-transfected into U2OS cells. The cells were imaged at 16 h post-transfection, a time point that yielded relatively low expression levels. Scale bar, 2 µm. (C) HeLa cells were transfected where indicated with plasmids encoding either monomeric GFP fused to a C-terminal region (“CTR”) of human Sec16A (residues 1909–2332), or an N-terminally triple-FLAG-tagged cytosolic domain (“Cyt”) of human Sec12 (residues 1–386). At 24 h post-transfection, the cells were lysed and the lysate was subjected to immunoprecipitation (“IP”) with anti-FLAG antibody. The immunoprecipitated material and 5% of the lysate (“5% Input”) was subjected to SDS-PAGE followed by immunoblotting with either anti-FLAG or anti-GFP antibody.

Concentration of Sec12 at tER sites has only been described in *P. pastoris*, so an obvious question is whether the Sec12–Sec16 interaction in this yeast is of broader significance. To address this issue, we revisited the localization of human Sec12. It was previously reported that human Sec12 was found throughout the ER [27], but the immunofluorescence data were ambiguous. We therefore repeated this experiment using an improved immunofluorescence protocol [28]. The labeling of human Sec12 was consistent with localization to the ER network, but punctate Sec12 labeling was also seen in tER sites (Fig. 8A) that contained Sec16A [17], which is the mammalian ortholog of yeast Sec16.

This result was confirmed by expressing GFP-tagged human Sec12. At various expression levels, GFP-Sec12 signal was present in the general ER but punctate structures were also visible (Fig. S2). As the expression level increased, the punctate structures became progressively larger (Fig. S2). This effect is reminiscent of the exaggerated tER sites seen with simultaneous overexpression of PpSec12 and PpSec16 in *P. pastoris* (see Figs. 1 and S1), except that in mammalian cells, overexpression of Sec12 alone suffices to generate enlarged structures. To verify that the punctate structures were tER sites, YFP-tagged Sec12 was co-expressed with CFP-tagged Sec16B, which is a tER-localized Sec16 homolog [17]. A large fraction of the YFP-Sec12 puncta overlapped with the CFP-Sec16B puncta at both low and high expression levels (Fig. 8B, and data not shown). We conclude that Sec12 is concentrated at tER sites in mammalian cells.

Sec16A contains a C-terminal region that appears to be related to the CTR of yeast Sec16 [17], suggesting that this domain of Sec16A may be responsible for recruiting human Sec12 to tER sites. As an initial test of this idea, we expressed in HeLa cells a FLAG-tagged cytosolic domain of human Sec12 together with a GFP-tagged C-terminal region of Sec16A. When the FLAG-tagged Sec12 fragment was immunoprecipitated, we reproducibly observed co-immunoprecipitation of a small amount of the GFP-tagged Sec16A fragment (Figure 8C). This result is consistent with a possible role of the C-terminal region of Sec16A in recruiting human Sec12 to tER sites.

## Discussion

The cytosolic domain of PpSec12 interacts with a tER-localized partner protein, and this interaction is readily reversible [12]. A likely candidate for the partner protein was Sec16, which binds to the cytosolic surface of the ER membrane at tER sites [8], [14]. Indeed, we have now obtained two lines of evidence that PpSec16 recruits PpSec12 to tER sites. The first line of evidence comes from overexpression studies in *P. pastoris*. When PpSec12 is overexpressed, most of the molecules are delocalized to the general ER because the tER-localized partner protein has been saturated [12]. We showed that simultaneous overexpression of PpSec16 suppresses the delocalization of overexpressed PpSec12, as would be expected if PpSec16 is the saturable partner protein. The second line of evidence comes from heterologous expression studies in *S. cerevisiae*. When PpSec12 is expressed in *S. cerevisiae*, it is found in the general ER, presumably because the interaction with ScSec16 is too weak to confer tER localization. We showed that simultaneous expression of PpSec16 in *S. cerevisiae* results in recruitment of PpSec12 to tER sites, consistent once again with the idea that PpSec16 is the partner protein for PpSec12.

In *S. cerevisiae*, the Sec12-like protein Sed4 binds to a C-terminal fragment of ScSec16 [24]. Similarly, we showed that PpSec12 binds to a C-terminal fragment of PpSec16. When this C-terminal fragment of PpSec16 is deleted, the truncated PpSec16 fails to recruit PpSec12 to tER sites either in *P. pastoris* or in *S. cerevisiae*. Biochemical pull-down experiments with recombinant proteins confirmed that a C-terminal fragment of PpSec16 can interact directly with the cytosolic domain of PpSec12. We found that the conserved C-terminal region (CTR) of PpSec16 is the major site of interaction with PpSec12, although a nonconserved stretch upstream of the CTR contributes to this interaction.

Deletion of the CTR is lethal [14], so an important question is whether the Sec12–Sec16 interaction is essential for life. Although we cannot yet answer this question with certainty, the data suggest that the Sec12–Sec16 interaction is nonessential. We found that PpSec12 binds weakly or not at all to ScSec16, and yet PpSec12 can replace ScSec12 in *S. cerevisiae*, even when *SED4* has been deleted to ensure that PpSec12 is the only Sec12 family member in the cells. Thus, the CTR probably has an essential function apart from binding Sec12. The CTR interacts with Sec23 in both yeast and mammalian cells [14], [17], so this Sec23 interaction may be the essential function of the CTR, with the Sec12 interaction playing a secondary role. This idea could be tested by identifying point mutations that selectively disrupt either the Sec12-CTR interaction or the Sec23-CTR interaction.

How conserved is the Sec12–Sec16 interaction? We showed here that Sec12 is concentrated at tER sites in human cells, and that the cytosolic domain of human Sec12 is capable of associating with the C-terminal region of human Sec16A. Recently, a Sec12–Sec16 interaction was also detected in *Caenorhabditis elegans* [29]. The combined data suggest that Sec12 has a conserved interaction with Sec16. This interaction may play a previously unsuspected role in tER organization, because exaggerated tER sites are seen upon simultaneous overexpression of PpSec12 and PpSec16 in *P. pastoris* or upon overexpression of human Sec12 in cultured mammalian cells.

Does the Sec12–Sec16 interaction modulate Sec12 function, or Sec16 function, or both? One possibility is that this interaction boosts the efficiency of COPII vesicle formation by concentrating Sec12 at tER sites, thereby enhancing local activation of Sar1 [30]. On the other hand, *S. cerevisiae* Sed4 binds Sec16 but lacks detectable guanine nucleotide exchange activity for Sar1 [24], [31], suggesting that the Sed4–Sec16 interaction serves to modulate Sec16 function. In support of this idea, overexpression of Sed4 suppresses temperature-sensitive *sec16* mutations [24]. We infer that Sed4 and Sec12 may share the ability to influence Sec16 function.

It is noteworthy that the CTR of Sec16 interacts both with Sec12, which is the guanine nucleotide exchange factor for Sar1, and with Sec23, which is the GTPase activating protein for Sar1. An intriguing question is whether the Sec12–Sec16 and Sec23–Sec16 interactions are mutually exclusive and perhaps antagonistic. Further biochemical and structural studies are needed to understand the complex interplay between Sec16 and the COPII pathway.

## Materials and Methods

### Fluorescence microscopy

Immunofluorescence microscopy was carried out as previously described [11], [12] except that the fixed cells were incubated in lyticase for 30 min at 30°C. For the yeast studies, samples were viewed with a Zeiss Axioplan 2 epifluorescence microscope using a 1.4-NA 100X Plan Apo objective, and images were captured with a Hamamatsu digital camera followed by processing in Adobe Photoshop to adjust brightness and contrast. For the mammalian cell studies, samples were viewed with a Zeiss LSM 510 META confocal microscope.

### Expression in *P. pastoris*

All *P. pastoris* strains were derivatives of PPY12 [32]. *P. pastoris* cells were transformed with linearized integrating vectors using electroporation [33]. The constructs used in this study are documented with annotated sequence files in Sequence Archive S1. Overexpression in *P. pastoris* was achieved using the strong methanol-inducible *AOX1* promoter as previously described [12]. Briefly, cells were grown overnight at 30°C in 5 mL of glycerol-containing SYG medium. This medium was then removed by filtration, and the cells were rinsed with methanol-containing SYM medium and then resuspended in SYM. After 8 h of growth with shaking in SYM, cells were either fixed and processed for immunofluorescence, or visualized directly by fluorescence microscopy. We showed previously that under these conditions, PpSec12 is overexpressed ∼190-fold [12].

Tagging of endogenous PpSec12 was achieved as follows. A BamHI fragment containing a modified 3′ portion of *PpSEC12* [12] was inserted into the BamHI site of pUC19-HIS4 [33] to create pLY051. This plasmid was then linearized with StyI and integrated at the *SEC12* locus via homologous recombination to tag PpSec12 with a Glu-Glu epitope tag, yielding strain EM19. Regulated expression of PpSec12 was achieved as follows. pEM04 was created from pIB4 [33] by replacing most of *P. pastoris HIS4* with *P. pastoris ARG4*. A gene encoding PpSec12-GG [12] was subcloned between the EcoRI and PstI sites of pEM04 to create pEM13. This plasmid was linearized by partial digestion with PflMI and then integrated at the *SEC12* locus to yield strain EM15, which has two tandem copies of *PpSEC12*: one copy that encodes PpSec12-GG under control of the endogenous promoter, and a second copy that encodes untagged PpSec12 under control of the *AOX1* promoter.

To label endogenous PpSec16 with monomeric enhanced GFP (mEGFP) [34], a PCR fragment spanning from codon 1178 of *PpSEC16* to ∼400 bp downstream of the stop codon was PCR amplified and inserted into the SmaI site of pUC19-HIS4 to create pME004. Then a fragment of *PpSEC16* fused to *mEGFP* was excised from pUC19-ARG4-S16C-mEGFP [8] using BspEI and XmnI, and inserted between the BspEI and SfoI sites of pME004 to create pME008. This plasmid was linearized with PshAI and integrated at the *SEC16* locus of strain EM15, yielding strain EM34.

Because full-length *PpSEC16* is toxic to *E. coli*, the *PpSEC16-GFP* overexpression construct was assembled by *in vitro* ligation as previously described [8] using plasmids pEM08, which contains the *AOX1* promoter followed by codons 1022–2550 of *PpSEC16* followed by *mEGFP*, and pEM12, which contains the *AOX1* promoter followed by codons 1–1678 of *PpSEC16*. A ligation product of fragments derived from pEM08 and pEM12 was integrated at the *HIS4* locus of strain EM15, yielding strain EM17. Two methods were used to create pEM08-type plasmids encoding deletions in a C-terminal fragment of PpSec16. In the first method, portions near the 3′ end of *PpSEC16* were deleted in pEM08 by primer-directed mutagenesis to create pEM48 (lacking codons 1967–2340), pEM49 (lacking codons 2341–2550), pEM50 (lacking codons 2501–2550), and pEM55 (lacking codons 2451–2550). In the second method, a pIB4 derivative was created in which codons 1505–1967 of *PpSEC16* were followed by *mEGFP* to create pEM43. pEM43, pEM48, pEM49, pEM50, and pEM55 were each used in an *in vitro* ligation reaction with pEM12, and the products were integrated at the *HIS4* locus of strain EM15, yielding strains EM16, EM25, EM26, EM27, and EM38, respectively.

Deletion of nonessential *PpSEC16* regions was accomplished by integrating linearized deletion constructs at the *PpSEC16* locus of strain EM19. This approach employed derivatives of plasmids pME005 and pLY100, as follows. pME005 was created by inserting codons 1178–2550 of *PpSEC16* plus ∼400 bp of downstream sequence into the SmaI site of pUC19-ARG4 [11]. Primer-directed mutagenesis of pME005 deleted codons 1967–2340 to create pEM54, which was linearized with PshAI for integration. Alternatively, a BstEII fragment spanning codons 682–1633 of *PpSEC16* was inserted into the BstEII site of pME005, and then primer-directed mutagenesis deleted codons 1010–1960 to create pLY080, which was linearized by partial digestion with XbaI for integration. pLY100 was created by inserting codons 1–1461 of *PpSEC16* plus ∼500 bp of upstream sequence into pUC19-ARG4. Primer-directed mutagenesis of pLY100 deleted either codons 500–648 to create pLY114, or codons 648–1010 to create pLY115, or codons 1–500 to create pLY116. These three plasmids were linearized with BssHII for gene replacement at the *PpSEC16* locus.

A strain expressing Sec13-DsRed as well as overexpressing PpSec12-GG and PpSec16-GFP was made as follows. The *KanMX* gene [35] was PCR amplified and inserted into the SspI site of pUC19 to create pUC19-KanMX. An EcoRI-XmnI fragment encoding a C-terminal portion of Sec13 fused to DsRed-Monomer was subcloned from pUC19-ARG4-Sec13-DsRed.M1 [8] into pUC19-KanMX to create pEM42. This plasmid was then linearized using MscI and integrated into the *SEC13* locus of strain EM17, yielding strain EM30.

### Expression in *S. cerevisiae*

All *S. cerevisiae* strains were derivatives of JK9-3d [36]. The constructs used in this study are documented with annotated sequence files in Sequence Archive S1. Overexpression in *S. cerevisiae* was achieved using the strong galactose-indicuble *GAL10* promoter [37]. Induction was carried out as follows. Cells were grown overnight in synthetic media containing dextrose. When the culture reached an OD_600_ of ∼0.4, the cells were transferred to synthetic media containing galactose, grown for 4.5 h, and then fixed and processed for immunofluorescence.

Heterologous expression of *P. pastoris* genes in *S. cerevisiae* was performed as follows. The starting strain carried a deletion of the endogenous *SEC12* gene, with the cells being kept alive by a 2μ *URA3* plasmid [38] encoding a Glu-Glu-tagged Sec12 protein under control of the *ScSEC12* promoter. This plasmid was either YEplac195-ScSec12-GG, which encodes *ScSEC12-GG*, or YEplac195-PpSec12-GG, which encodes *PpSEC12-GG* [12]. The chromosomal *ScSEC13* gene in these strains was then tagged with a triple-CFP cassette using pUC19-URA3-SEC13-CFPx3 that had been linearized with BstEII, yielding strains LY05 and LY06. Additional regulated expression of PpSec16-YFP was achieved using the *GAL10* promoter. Because the full-length *PpSEC16* gene is toxic to *E. coli*, the PpSec16-YFP plasmid was constructed by *in vitro* ligation of fragments from pEM45, which encodes a 5′ portion of *PpSEC16*, and pEM46, which encodes a 3′ portion of *PpSEC16* fused to a triple-YFP cassette. Strains LY03 and LY04 expressed ScSec13-3xCFP, PpSec16-3xYFP, and either ScSec12-GG or PpSec12-GG, respectively.

### GST pull-down and immunoblotting

To express GST fused to a C-terminal fragment of PpSec16, codons 1960–2550 of *PpSEC16* were subcloned between the XmaI and XhoI sites of pGEX-4T-1 (GE Healthcare) to create pME107. To express the cytosolic domain of PpSec12 fused to a hexahistidine tag, codons 1–337 of PpSEC12 were amplified by PCR and inserted between the NdeI and XhoI sites of pET21a(+) (Novagen), yielding pET21a(+)-PpSec12(cyto). pME107 and pET21a(+)-PpSec12(cyto) were each transformed into *E. coli* Rosetta cells (Novagen) containing the pLysS plasmid. For protein expression, a 50-mL culture of cells was grown at 37°C with shaking for 5 h, and then protein expression was induced with 1 mM isopropyl-β-D-thiogalactopyranoside for a further 4 h.

Cell lysis and GST pull-down were conducted using the Thermo Scientific Pierce ProFound Pull-Down GST Protein∶Protein Interaction kit. A brief protocol follows. All incubations and washes were performed using a 1∶1 mixture of ProFound Lysis Buffer and 1 M Tris-buffered saline. Cell pellets were lysed for 30 minutes in the presence of Benzonase (Novagen), and lysates were clarified by centrifugation at 100,000×g to obtain the Input fraction. Glutathione-agarose beads were incubated for 1 h at 4°C with clarified lysate from cells expressing GST-Sec16(1960–2550). The beads were washed, and then incubated for 1 h at 4°C with clarified lysate from cells expressing PpSec12(cyto)-His6. At this point the beads were spun in a column and the flow-through was collected as the Unbound fraction. The beads were then washed, followed by a 10-minute incubation at room temperature with 100 mM glutathione to elute the Bound fraction.

Equivalent amounts of the Input, Bound, and Unbound fractions were boiled in SDS-PAGE sample buffer and loaded onto an SDS-PAGE gel. Separated proteins were transferred to PVDF membranes, and the hexahistidine-tagged PpSec12 cytosolic domain was detected using a 6-His monoclonal antibody (Covance, catalog no. MMS-156P) and the Supersignal West Femto kit (Pierce).

### Plasmid shuffle experiment

pEM60 was made by ligating a PCR product containing *SED4*, including the promoter and terminator, into the *CEN URA3* plasmid YCplac33 [38]. pEM61 and pEM62 were created by subcloning *ScSEC12-GG* or *PpSEC12-GG*, respectively, together with the *ScSEC12* promoter [12] into the 2 µm *LEU2* plasmid YEplac181 [38].

Strains EM59 to EM62 were created by first deleting one copy of the *SED4* coding sequence in a diploid strain in which one copy of *ScSEC12* had been replaced by *KanMX* [12]. To delete *SED4*, the cells were transformed with a PCR product containing a hygromycin B resistance gene [39] flanked by sequences upstream and downstream of the *SED4* coding sequence, with selection on rich medium containing 200 µg/mL hygromycin B (US Biologicals). Next, pEM60 plus either pEM61 or pEM62 were introduced to create strains EM54 and EM55, respectively. These strains were then sporulated and subjected to tetrad dissection. A sporulation defect was observed, with two- and three-spore tetrads outnumbering four-spore tetrads, probably reflecting a role for *SED4* during sporulation [40]. Haploid clones were tested on selective media to assess the presence of plasmids and drug resistance markers.

Survival in the absence of *SED4* was tested as follows. Strains EM59 to EM62, plus a control strain in which a *ScSEC12* deletion was rescued by YEplac195-ScSEC12-GG, were grown to mid-log phase in rich medium overnight to give the cells an opportunity to lose the *URA3* plasmids. Equal numbers of cells were then spotted onto plates containing 1 g/L 5-FOA (US Biologicals). Plates were incubated in the dark at 30°C for several days to allow for growth of colonies lacking the *URA3* plasmids.

### Analysis of mammalian Sec12 and Sec16

For immunofluorescence, human Sec12 was detected using an affinity purified goat anti-human PREB antibody from R&D Systems (cat. #AF5557, diluted 1∶50), and Sec16A was detected using a rabbit polyclonal anti-KIAA0310 antibody from Bethyl Laboratories (cat. #BL2467, diluted 1∶50). These primary antibodies were detected with Alexa Fluor 594 donkey anti-goat and Alexa Fluor 488 chicken anti-rabbit IgG secondary antibodies (Invitrogen), respectively. For immunoblotting, GFP was detected using a rabbit polyclonal antibody (Abcam cat. #ab290, diluted 1∶1000), and the FLAG epitope was detected using a mouse monoclonal antibody (Sigma cat. #F1804, diluted 1∶2000).

For expression of fluorescently tagged proteins in U2OS cells, full-length human Sec12 was N-terminally tagged with monomeric enhanced GFP (mEGFP) or YFP (mEYFP), and full-length Sec16B was N-terminally tagged with monomeric enhanced CFP (mECFP) [34]. The human Sec12 and Sec16B genes were amplified from cDNAs by PCR and subcloned into pEGFP-C1 (Clontech) downstream of the EGFP gene, followed by replacement of EGFP with either mEGFP or mEYFP or mECFP. Sec16B is identical to the gene that we previously designated Sec16S [17]. These constructs are documented with annotated sequence files in Sequence Archive S1.

For co-immunoprecipitation, tagged fragments of human Sec12 and Sec16A [17] were expressed transiently from the CMV promoter. The cytosolic domain of human Sec12 (codons 1–386) was amplified from a cDNA by PCR and subcloned into pCMV-3FLAG-1A (Stratagene) downstream of a triple-FLAG cassette. A C-terminal region of Sec16A (codons 1909–2332) was amplified from a cDNA by PCR and subcloned into pmEGFP-C1, which is a derivative of pEGFP-C1 (Clontech) carrying the monomerizing A206K mutation [34]. In the case of Sec16A, which we previously designated Sec16L [17], our original description of the gene overlooked the first 178 codons [18], so we added those codons for the numbering used here. Because our cDNA lacks the 75-bp intron between the 28th and 29th exons [17], our numbering for the C-terminal fragment of Sec16A differs by 25 amino acids from the numbering used elsewhere [18]. These constructs are documented with annotated sequence files in Sequence Archive S1. Plasmids were transfected into HeLa cells using calcium phosphate transfection. The cells were harvested at 24 h post-transfection, and lysed in 50 mM Tris-HCl, pH 7.9, 50 mM NaCl, 1 mM phenylmethylsulfonyl fluoride, 0.5% NP-40 supplemented with the Complete mini protease inhibitor cocktail (Roche). Approximately 3 mg of cell lysate was used for each immunoprecipitation. Protein A-agarose beads (Calbiochem) were pre-blocked with 1% bovine serum albumin and then incubated at 4°C with polyclonal rabbit anti-FLAG antibody (Sigma). Immunoprecipitation was performed overnight at 4°C. Then the beads were washed three times with 20 mM Na^+^-HEPES, pH 7.9, 100 mM NaCl, 0.1% NP-40. Finally, bound material was eluted by boiling in SDS-PAGE sample buffer, followed by SDS-PAGE, transfer to nitrocellulose, and immunoblotting [17].

## Supporting Information

Figure S1 **Colocalization of PpSec16 with PpSec13 in** ***P. pastoris*** **cells overexpressing both PpSec12 and PpSec16.** The method of Fig. 1 was used to achieve simultaneous overexpression of PpSec12 and GFP-tagged PpSec16. In addition, PpSec13 was tagged with DsRed by gene replacement. The overexpressed PpSec16-GFP colocalized with PpSec13-DsRed, confirming that the structures labeled with PpSec16-GFP were exaggerated tER sites. Scale bar, 2 µm.
(TIF)

Figure S2 **Localization of GFP-tagged human Sec12 at different expression levels.** A plasmid encoding GFP-tagged full-length human Sec12 was transfected into U2OS human osteosarcoma cells. The cells were imaged at either (A) 12 h, (B) 24 h, or (C, D) 36 h post-transfection. Representative images are shown for cells expressing GFP-Sec12 at (A) low, (B) moderate, (C) high, and (D) very high levels. These cells were imaged at different exposure levels according to their fluorescence intensities. As the expression level of GFP-Sec12 increased, the punctate structures became progressively larger. Scale bar, 5 µm.
(TIF)

Sequence Archive S1 **Annotated sequence files for the constructs used in this study.** A compressed folder contains GenBank-style sequence files for the relevant constructs.
(RAR)
