# Supplementary figures and images for: Sec12 Binds to Sec16 at Transitional ER Sites
Source: PLoS One. 2012 Feb 8;7(2):e31156. doi: 10.1371/journal.pone.0031156 (PMC3275590; doi:10.1371/journal.pone.0031156)

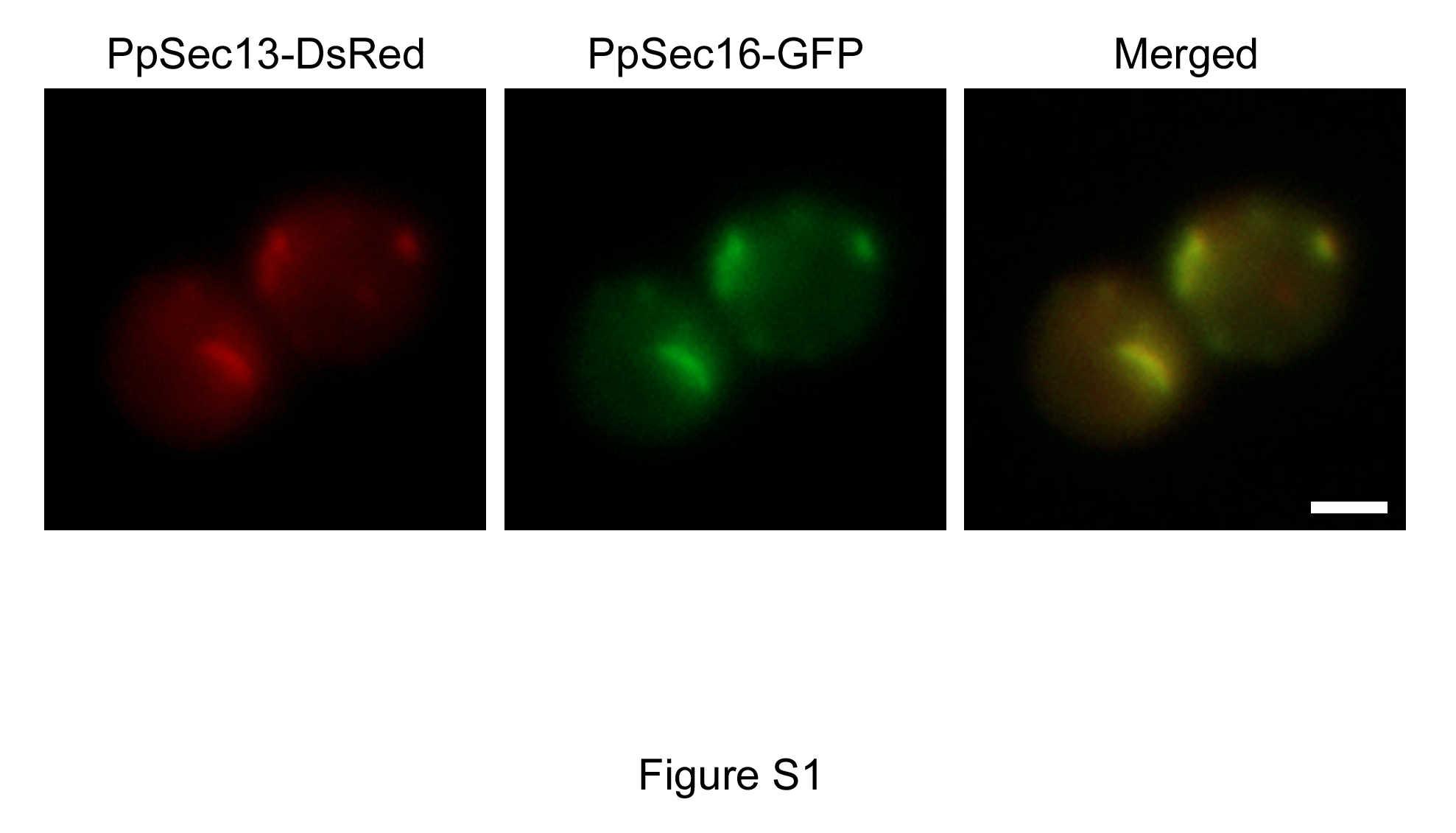

Supplement: Figure S1 — Colocalization of PpSec16 with PpSec13 in P. pastoris cells overexpressing both PpSec12 and PpSec16. The method of Fig. 1 was used to achieve simultaneous overexpression of PpSec12 and GFP-tagged PpSec16. In addition, PpSec13 was tagged with DsRed by gene replacement. The overexpressed PpSec16-GFP colocalized with PpSec13-DsRed, confirming that the structures labeled with PpSec16-GFP were exaggerated tER sites. Scale bar, 2 µm. (TIF) [file pone.0031156.s001.tif]

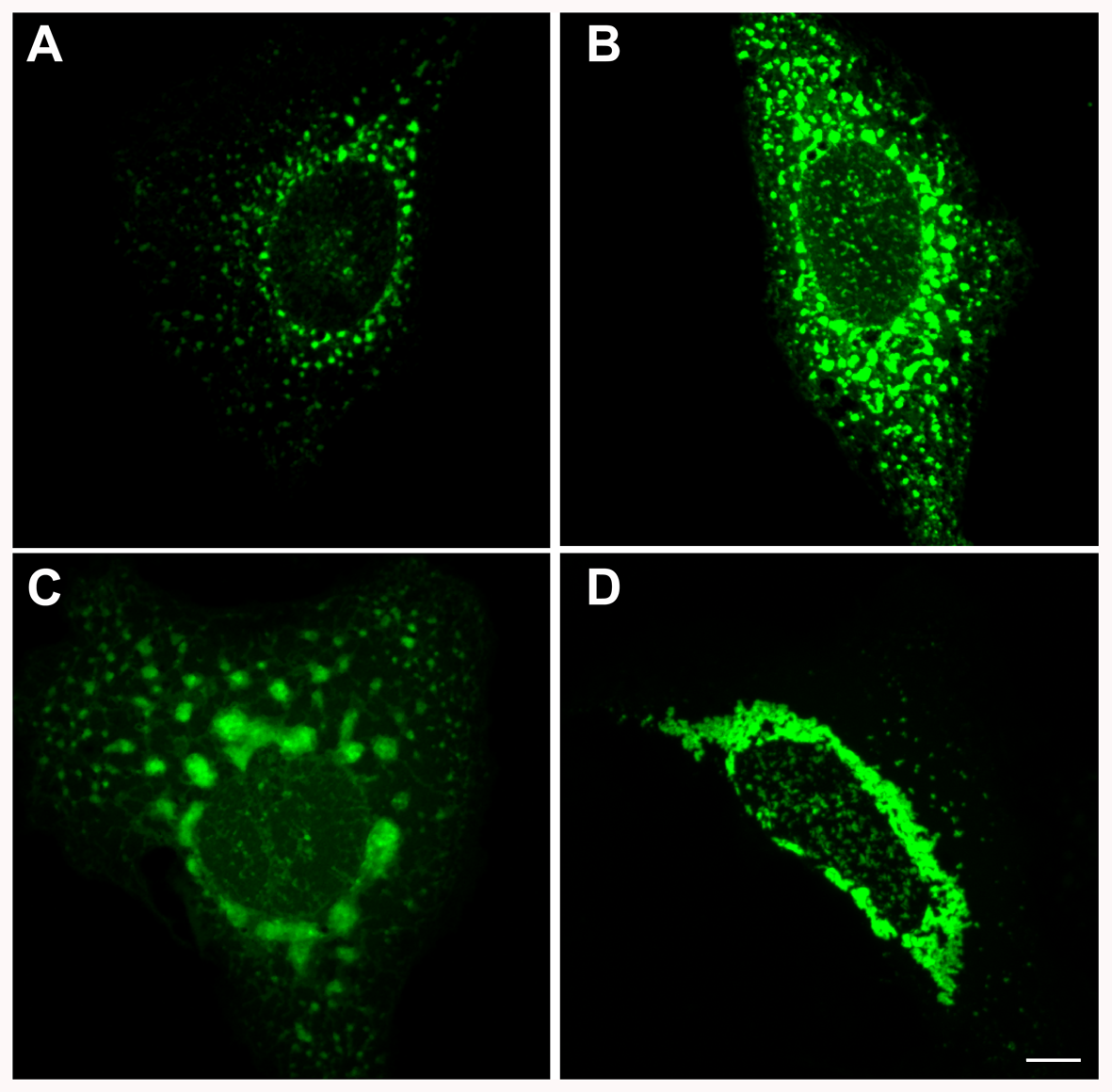

Supplement: Figure S2 — Localization of GFP-tagged human Sec12 at different expression levels. A plasmid encoding GFP-tagged full-length human Sec12 was transfected into U2OS human osteosarcoma cells. The cells were imaged at either (A) 12 h, (B) 24 h, or (C, D) 36 h post-transfection. Representative images are shown for cells expressing GFP-Sec12 at (A) low, (B) moderate, (C) high, and (D) very high levels. These cells were imaged at different exposure levels according to their fluorescence intensities. As the expression level of GFP-Sec12 increased, the punctate structures became progressively larger. Scale bar, 5 µm. (TIF) [file pone.0031156.s002.tif]
